# Pituitary Adenoma Apoplexy in an Adolescent: A Case Report and Review of the Literature

**DOI:** 10.4274/jcrpe.4420

**Published:** 2017-09-01

**Authors:** Hero Zijlker, Sebastian Schagen, Jan Maarten Wit, Nienke Biermasz, Wouter van Furth, Wilma Oostdijk

**Affiliations:** 1 Leiden University Medical Center, Department of Pediatrics, Leiden, The Netherlands; 2 Leiden University Medical Center, Department of Medicine, Division of Endocrinology, Leiden, The Netherlands; 3 Leiden University Medical Center, Department of Neurosurgery, Leiden, The Netherlands

**Keywords:** pituitary adenoma, apoplexy, panhypopituitarism, adolescents, pituitary abscess, headache, magnetic resonance imaging

## Abstract

We present a 13-year-old boy who was admitted with complaints of a state of progressive sleepiness and a sudden headache with vomiting and fever. Laboratory testing showed hypoglycemia, multiple pituitary hormonal deficiencies, and an elevated C-reactive protein level. A cranial magnetic resonance imaging (MRI) showed an opaque sphenoid sinus and an intrasellar mass suggesting hemorrhage, so that we suspected pituitary apoplexy (PA) originating from a non-functioning adenoma, although a pituitary abscess could not completely be excluded. The boy was treated with antibiotics, hydrocortisone, and levothyroxine. Due to his rapid clinical improvement, no surgery was performed and we considered the diagnosis of PA as confirmed. At follow-up, the MRI scan showed a small residual lesion. Pituitary deficiencies of growth hormone, adrenocorticotropic hormone (ACTH), thyroid-stimulating hormone, and vasopressin persisted. A literature search of all well-documented cases of PA in children or adolescents (n=30, 13 boys and 17 girls) indicated that this condition is rare below 20 years of age but must be considered when a patient experiences headache with or without visual disturbances, even in the presence of clinical and laboratory signals suggestive of pituitary abscess. MRI neuroimaging is helpful in the differential diagnosis. In both conditions, the possibility of ACTH deficiency should always be considered, investigated, and treated. In cases without severe neuro-ophthalmological deficits and/or with a rapid and positive response to acute medical management, one can abstain from surgical treatment.

**What is already known on this topic?**

Pituitary apoplexy (PA) is a rare clinical syndrome in adolescents that can cause a life-threatening situation. PA is frequently seen in non-functioning adenomas and often results in headache and visual impairments.

**What this study adds?**

Our study is to help physicians in differentiating between a PA and a pituitary abscess, to create an overview of the possible clinical symptoms seen in PA, and to create awareness for a possible adrenocorticotropic hormone-deficiency.

## INTRODUCTION

Neoplasms of the pituitary gland are extremely rare in childhood and adolescence (1:1.000,000) (1). Of all pituitary neoplasms, less than 10% are diagnosed in children and adolescents. Most of these are craniopharyngiomas (80-90%) and relatively few (3% of all intracranial neoplasms) are adenomas. Of all adenomas in patients younger than 20 years, approximately 97% secrete hormones and 16% develop pituitary apoplexy (PA) (2).

PA is a clinical syndrome caused by hemorrhage or infarction of the pituitary gland and is predominantly seen in patients with pituitary adenomas, probably due to their relatively high metabolism, limited blood flow, and high intratumoral pressure when compared to other primary central nervous system tumors (3). PA occurs relatively often in large macroadenomas (4). Since non-functioning adenomas (NFAs) are on average larger than endocrine active adenomas, PA is relatively more frequently observed in NFAs (2,4). The presenting symptoms include sudden and severe headache, visual disturbances, and various neurological signs (4). In adults, PA is more common in males between 50-69 years and precipitating factors include angiography, cardiac surgery, anticoagulant therapy, and dynamic hormonal testing or gonadotropin-releasing hormone (GnRH) agonist treatment. However, little is known about this condition in patients younger than 20 years.

In 1972, Dawson and Kothandaram (5) were the first to describe an adolescent with PA. Since then, only a few case reports and individual cases extracted from larger case series have been reported in the literature. In 2015, Jankowski et al (6) presented a case series of nine adolescents with PA, comparing symptomatology, neuroimaging, pathology, and outcomes to those in adults.

In this paper, we report a case of a 13-year-old boy presenting with sudden and severe headache. Additional investigations suggested PA originating from an non-functioning adenoma (NFA), although initially a pituitary abscess [accounting for less than 1% of all pituitary lesions (7)] could not be completely excluded. We reviewed the literature and summarize the clinical, biochemical, and imaging characteristics of all reported cases of PA in patients younger than 20 years. Pediatricians should be aware that this condition which is frequently accompanied by adrenocorticotropic hormone (ACTH) deficiency, although extremely rare, can occur in children and adolescents and that the differential diagnosis with pituitary abscess can be difficult.

## CASE REPORT

A 13-year-old boy, with an uninformative previous medical history, presented at the pediatric clinic of a general hospital with complaints of severe fatigue which had lasted for several months. Four days prior to admission, he had become progressively sleepy and experienced a sudden and severe stabbing frontal headache with vomiting and phonophobia. At physical examination, he had a normal level of consciousness, fever up to 38 degrees Celsius, and no neurological abnormalities. He had no visual disturbances and normal extra-ocular movements. His linear growth and pubertal development had been unremarkable; at admission, his height standard deviation score (SDS) was 0.0 (8). His body mass index was 20 kg/m^2^ (+1.0 SDS) (9) and Tanner stage was G3P3A2 with testes of 12 mL (assessed with the Prader orchidometer). Initial laboratory results demonstrated a normal white blood cell count (9.2x10^9^ /L; 54% neutrophils), an elevated serum C-reactive protein (CRP) (201 mg/L) and hypoglycemia (2.8 mmol/L). Despite normal neurological examination, increased intracranial pressure due to a brain tumor or abscess was considered because of the severe headache and vomiting. For this reason, broad-spectrum antibiotics were immediately administered intravenously and he was referred to our academic hospital.

**Figure 1:**
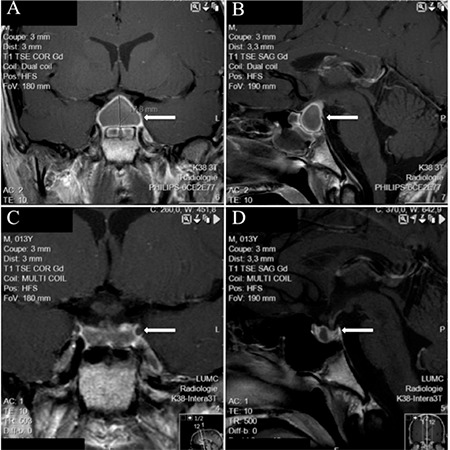
Magnetic resonance imaging: T1-weighted imaging. At presentation: coronal (A) and sagittal (B) view showing rim enhancement and sphenoid sinus mucosal thickening. Three months later: coronal (C) and sagittal (D) view showing substantial mass reduction

Magnetic resonance imaging (MRI) of the cerebrum showed a sellar mass with suprasellar extension (2.5x2.0 cm) and slight optic chiasm compression (Figure 1). The mass appeared heterogeneously hyperintense on T1-weighted imaging (T1WI) and hypointense to isointense on T2-weighted imaging (T2WI). Also sphenoid sinus mucosal thickening and rim enhancement of the mass after gadolinium contrast were noted. These MRI findings were highly suggestive of hemorrhage that most likely originated from a pre-existing pituitary adenoma.

The endocrine investigations (Table 1) demonstrated central hypothyroidism, hypocortisolism, and hypogonadism, as well as low serum insulin-like growth factor (IGF)-I and IGF binding proteins-3 levels suggestive of growth hormone (GH) deficiency. A stress dose of hydrocortisone was immediately administered followed by substitution with hydrocortisone and levothyroxine. We considered PA originating from an NFA most likely, based on the specific MRI findings.

Because we could not completely exclude pituitary abscess, immediate surgical intervention was considered. However, rapid clinical improvement was noted after administration of broad-spectrum antibiotics, and hydrocortisone was started. One day after initiation of treatment, the fever disappeared and CRP levels gradually declined to 48 mg/L on the fourth post-treatment day. CRP levels were completely normalized after 2 weeks. Surgery was eventually not performed due to the boy’s rapid clinical improvement, the sella enlargement, which is unusual for pituitary abscess, and the sphenoid sinus mucosal thickening seen on the MRI, which suggested a possible infectious process on the surgical route. The combination of MRI findings and clinical course has made the diagnosis of PA virtually certain, although not histologically confirmed. Remarkably, diabetes insipidus (DI) developed three days after admission despite conservative treatment.

The patient was discharged after five days with oral broad-spectrum antibiotics, hydrocortisone, levothyroxine, and vasopressin. A small residual lesion was seen on the MRI three months later (Figure 1), which resolved after 6 months. The thyroid-stimulating hormone (TSH) and ACTH deficiencies persisted. GH deficiency was diagnosed by a very low (GH peak 0.9 ug/L) response to GH stimulation tests, so that GH substitution was started. The pituitary-gonadal axis was not affected (normal pubertal GnRH test) and puberty progressed normally. Genetic evaluation showed no abnormalities in the MEN1 gene.

### Literature Search

A literature search was performed in databases Pubmed, Embase, Web of Science, Medline, and Cochrane to identify all cases of PA originating from an adenoma in patients younger than 20 years. Only publications written in English were included.

Cases found in larger case series with no or scarce individual descriptions were excluded (n=36) (2,10,11). This resulted in 30 cases published between 1972 and 2016: 16 case reports and 14 cases extracted from 6 case series (Table 2) (5,6,11,12,13,14,15,16,17,18,19,20,21,22,23,24,25,26,27,28,29,30). The total group consisted of 13 boys and 17 girls with a mean age of 15.3 years (range 6-19 years, median 16 years).

## DISCUSSION

PA in children and adolescents is a rare entity that requires rapid and adequate treatment to prevent a life-threatening situation. Based upon our literature search, various aspects are discussed.

### Non-Functioning Pituitary Adenomas and Pituitary Apoplexy

Only 3% of all pituitary adenomas in patients younger than 20 years are NFAs (2). This low percentage can be explained by the slow growth of pituitary adenomas and data suggesting that 85-90% of normal pituitary gland and optic chiasm have to be compromised to develop endocrine insufficiencies and visual deficits, respectively (31).

So far, only four extensively described cases of patients younger than 20 years with PA originating from a NFA have been published (13,18,22,23). Based upon these data, we conclude that PA originating from a NFA is an extremely rare entity in this age group, despite the fact that PA is likely to occur in NFAs.

### Adrenocorticotropic Hormone Deficiency

A corticotropic deficiency can lead to serious hemodynamic instabilities causing a life-threatening situation and this was seen in 50-80% of the adult patients with PA (4). No accurate data are available for determining the prevalence of corticotropic deficiencies in children and adolescents with PA. However, 43% (5,12,13,14,16,17,18,19,22,23,25,27,29) of the reported cases received steroid replacement therapy, indicating that corticotropic deficiency is commonly seen in young patients with PA. Even if serum cortisol and its response to an ACTH injection appear normal in the acute situation, an ACTH deficiency can become apparent in subsequent days.

Due to the severity of a possible hemodynamic instability seen by an Addisonian crisis, every patient with signs or symptoms of PA should immediately be treated with steroids (4). Our patient immediately received hydrocortisone after hypocortisolism was noticed. In the following days, his clinical condition improved rapidly. However, since antibiotics were also administered, it was hard to say which component of the treatment led to his improvement.

### Differentiation Between Pituitary Apoplexy Originating from Adenoma and Pituitary Abscess

The differentiation between PA and pituitary abscess is of vital importance because of the contrasting therapeutic consequences: pituitary abscess is an indication for immediate operation, while an expectative policy would usually be the best option in patients with PA. Based on the literature, four elements are discussed for the differential diagnosis between PA and pituitary abscess: 1) Clinical presentation, 2) Infection parameters, 3) Endocrine function, 4) Neuroimaging.

### Clinical Presentation

Liu et al (7) described the largest series (33 patients; mean age 42 years; range 12 to 63 years) of patients with pituitary abscess so far. In their cohort, the most common clinical symptoms were headache (70%), with no common pattern, and visual disturbances (27%). Headache with a sudden onset can also occur in patients with pituitary abscess (32). In the reports on young individuals we reviewed, headache was also the most common described symptom (90%), with a sudden onset in one third of them. Despite the frequently described sudden onset, no common pattern of headache was observed. Visual disturbances were described in 73% of cases.

Regarding the strong clinical similarities between PA and pituitary abscess, no differentiation between these two conditions could be made based on clinical presentation.

Furthermore a low ‘PA Score’ (0 out of 10 points), based on the absence of visual symptoms and his normal level of consciousness (33), was found in our patient. This clinical tool did not help us differentiate the two conditions.

### Infection Parameters

Initial laboratory results demonstrated a normal white blood cell count, an elevated serum CRP, and hypoglycemia in our patient, which together with the elevated body temperature was initially considered highly suggestive of pituitary abscess. However, in the literature, a 57-year-old man was described with a presentation similar to that of our patient (34). In that patient, there was a strong suspicion of bacterial meningoencephalitis due to the combination of fever, meningeal irritation, elevated CRP (109 mg/L), and neutrophilic leukocytosis (13.600/mm^3^, 66% neutrophils). MRI imaging showed a sellar mass which initially was defined as a secondary pituitary abscess. However, cerebrospinal fluid contained no microbes, and during surgery, biopsy was obtained that demonstrated PA originating from an adenoma. Also, the presence of fever appears to have a low discriminative power, since this was reported in one third of the cases in young individuals with PA in 18% of patients with pituitary abscess (7). Despite the difference in age in comparison with our patient, we conclude that a very elevated CRP, leukocytosis, and fever can also occur in PA.

### Endocrine Function

Our patient’s endocrine analysis demonstrated hypopituitarism. This endocrine condition was reported in 26% of the reviewed cases and in 85% of cases with pituitary abscess (7). In patients with pituitary abscess, 70% presented with DI (7). In contrast, less than 5% of the cases with PA described by Briet et al (4) presented with DI and none of the reviewed were young cases. DI is a condition that usually develops after pituitary surgery (35). It is noteworthy that DI may be masked by secondary adrenal failure and develop after steroid or thyroid replacement. From the reviewed cases, seven (23%) developed DI - five of them were operated and 2 were treated conservatively (Table 3). Persistent DI was described in 3 cases and transient - in 1. Description of DI in the other 3 cases is inconclusive. Eight of nine cases originally described by Jankowski et al (6) are included in our study. Four of them developed transient DI and 1 developed persistent DI. DI after conservative treatment of PA has sporadically been documented in the literature. Only two non-surgically treated cases included in our group developed DI (13,18).

We conclude that endocrine function tests are necessary but can hardly assist in differentiating PA from pituitary abscess.

### Neuroimaging

The MRI showed a space-occupying lesion at the site of the sella turcica in our patient.

The typical cystic features of pituitary abscess are hypointense on T1WI but hyperintense on T2WI. Rim enhancement can be seen after gadolinium in 64% of pituitary abscesses (7). The remaining 36% had hypointense to isointense signaling on T1WI and isointense to hyperintense signaling on T2WI. The MRI performed in our patient showed a heterogeneous hyperintense signal on T1WI, hypointense to isointense signal on T2WI with rim enhancement after gadolinium, as well as sphenoid sinus mucosal thickening. These T1WI and T2WI features were consistent with a hemorrhage (36), although rim enhancement after gadolinium is more often seen in pituitary abscess (64%) than in PA (36%).

In our reviewed young patients, all MRI findings were suggestive of hemorrhage within an adenoma. Also, compression of the surrounding structures was stated in 15 cases, involving the optic chiasm in 14, the infundibulum in 4, and the hypothalamus in one. Furthermore, sphenoid sinus mucosal thickening was observed in our patient, a finding that could suggest an inflammation of the sinus and may constitute a potential cause of a secondary pituitary abscess (37,38). However, mucosal thickening was also seen in 2 of the 9 adolescent patients with PA investigated by Jankowski et al (6) and is probably due to venous engorgement secondary to PA. Thus, when a patient has either PA or pituitary abscess, the differentiation should be made by MRI neuroimaging. Clinical presentation, PA Score, infection parameters, and endocrine function are not helpful in the differentiation of these two conditions. In our patient, based on the MRI neuroimaging findings, PA was more likely than pituitary abscess.

### Treatment

We did not find any publication comparing the outcome of different therapeutic strategies in patients with PA younger than 20 years. In adults, Singh et al (39) analyzed the outcomes of case series of different treatment options of PA (57 males; 30 females; mean age 51 years; range 15 to 91 years). They concluded that the outcome of most patients was excellent and that no statistically significantly differences existed between the surgically and conservatively treated patients. All patients with endocrine deficiencies or electrolyte disturbances were acutely managed with hormonal and electrolytes substitution. Most of the patients who received early surgery (surgery within a median time of 5 days, range 3 to 10 days) had severe neuro-ophthalmological deficits at presentation. On the other hand, patients who lacked severe neuro-ophthalmological deficits, including patients with reduced consciousness, or patients with a rapid response to acute management, were adequately managed conservatively. In line with this advice, our patient was not subjected to a surgical intervention because of his rapid clinical improvement with antibiotics, hydrocortisone, and levothyroxine substitution.

Out of the 30 reported young cases, 23 underwent surgery, mostly via the transsphenoidal route (78%). Nine of these patients received endocrine replacement therapy. Of these 9 patients, 1 died shortly after surgery and there was no mention of symptom relief in 5. Seven cases were managed conservatively, of whom 6 received endocrine replacement therapy.

Our patient received prolonged endocrine replacement therapy due to persisting hormonal deficiencies and this was also seen in 15 reported cases (50%), illustrating the high risk of permanent damage of the pituitary gland caused by PA.

## CONCLUSIONS

PA is a rare condition seen in patients younger than 20 years, but must be considered when a patient experiences headache with or without visual disturbances, even in the presence of clinical or laboratory findings suggestive of an infection. There should be a high index of suspicion for ACTH deficiency which must be promptly treated with stress doses of hydrocortisone. Differentiation between pituitary abscess and PA is difficult. Type of headache, elevated CRP, endocrinological status, and fever do not differentiate between the two conditions. MRI neuroimaging is helpful in making the diagnosis since differences exist in T1W1 and T2W1 images of patients with hemorrhage and abscess. We agree with Singh et al (39) that without severe neuro-ophthalmological deficits or with a quick response to the acute management, patients can be treated conservatively. Furthermore, multiple persistent pituitary deficiencies, including DI, are a common outcome.

## Figures and Tables

**Table 1:**
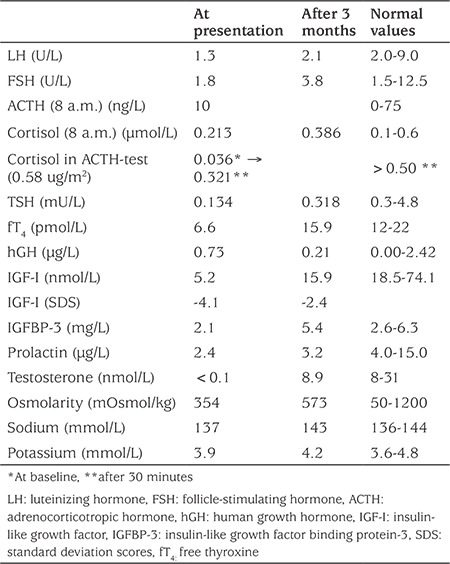
Hormonal values of our case at presentation and 3 months later

**Table 2:**
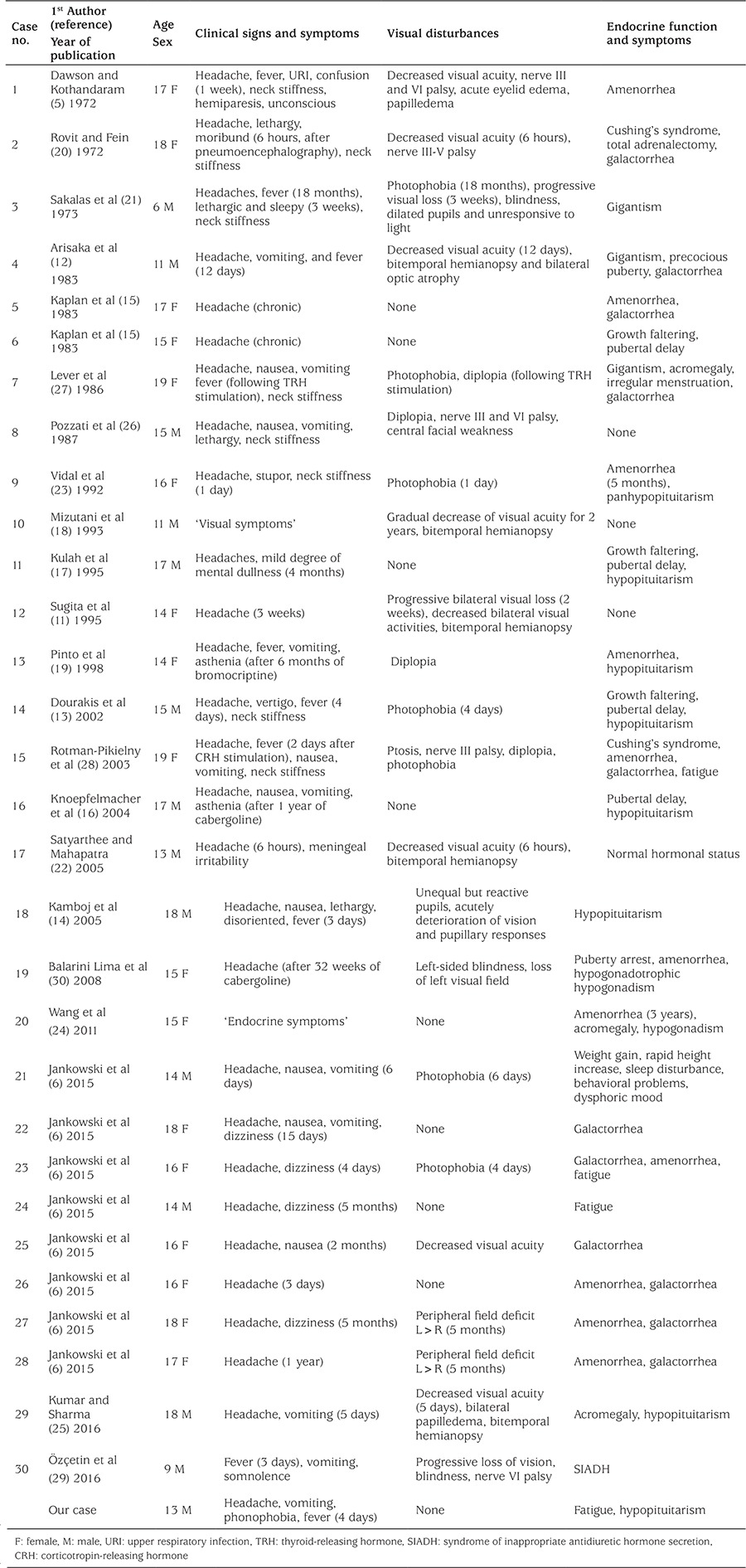
Summary of clinical and pathological characteristics of cases younger than 20 years with pituitary apoplexy

**Table 3:**
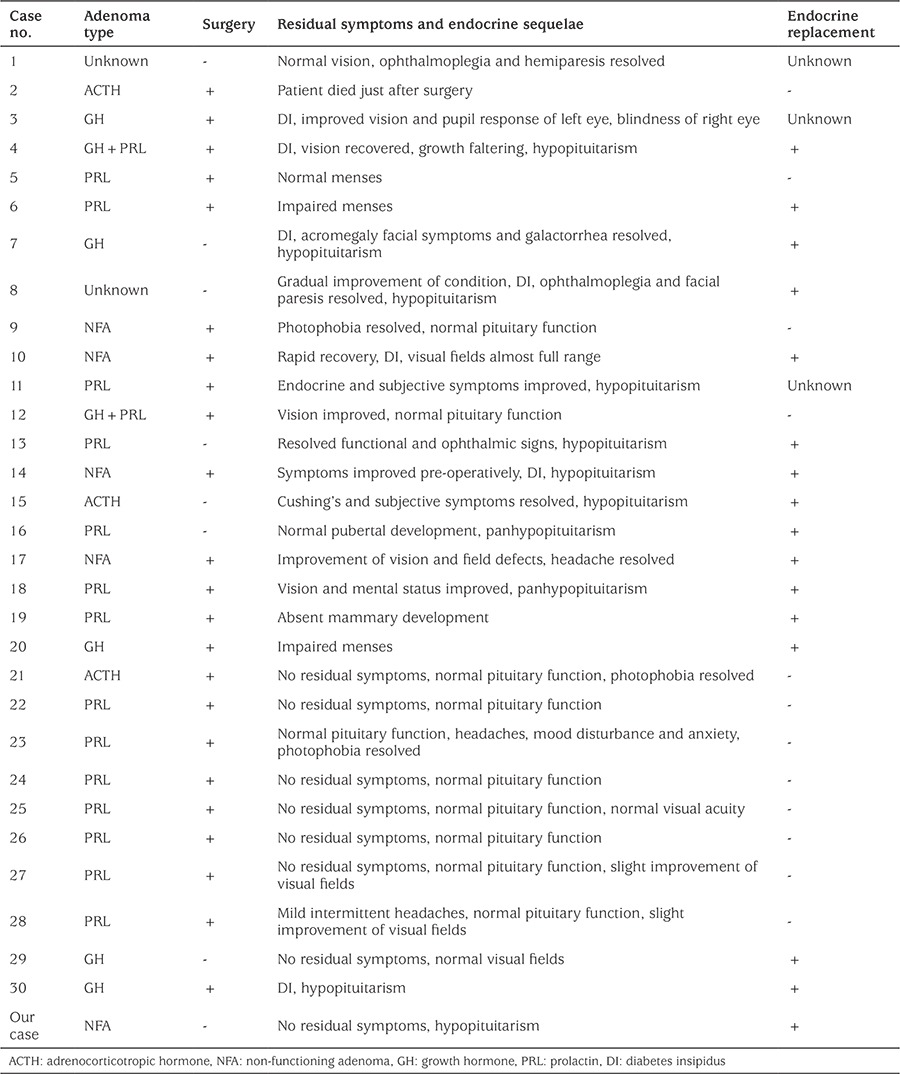
Summary of pathological characteristics, treatment and follow-up data of cases younger than 20 years with pituitary apoplexy
